# Supplementary material for: Innate Immunity and Sex: Distinct Inflammatory Profiles Associated with Murine Pain in Acute Synovitis
Source: Cells. 2023 Jul 22;12(14):1913. doi: 10.3390/cells12141913 (PMC10378550; doi:10.3390/cells12141913)
Supplement: Supplementary file 1 [file cells-12-01913-s001.zip › Figure S1 and Table S1-S3.pdf]

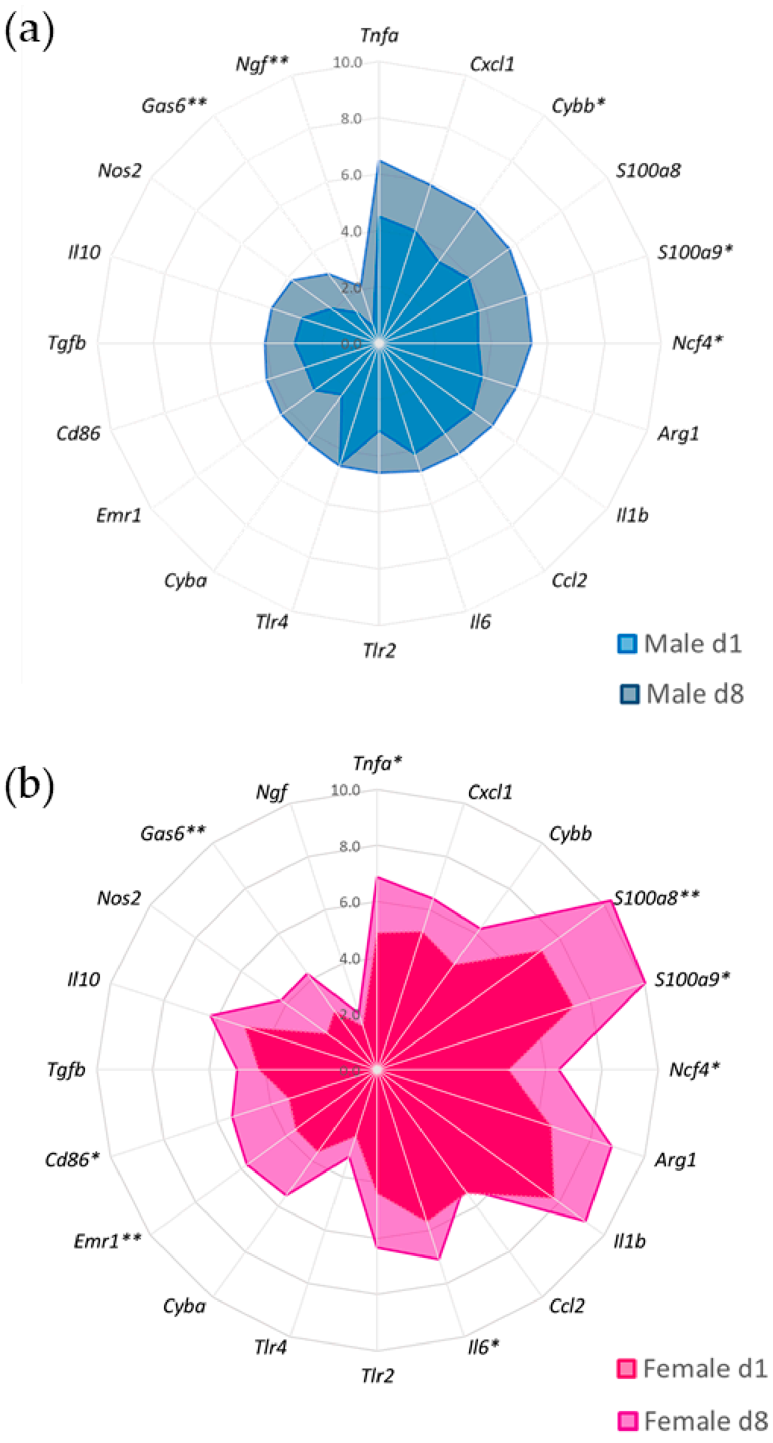

Figure S1. Synovial gene expression following SCW intraarticular injections showed more robust inflammatory response during pre-sensitized response. Spider chart based on the synovial genes expressed (ddCt) during acute SCW arthritis based on sex: (a) males day 1/8 and (b) females day1/8. Genes were sorted by stronger gene expression (ddCt) based on the male d8 group. Significance was calculated compared between sexes within first or second SCW response using Two-way ANOVA with Šídák's multiple comparisons test. N=7 males and 8 females per SCW group. *$p < 0.05$, **$p \leq 0.01$, ***$p \leq 0.001$ and ****$p \leq 0.0001$.

Table S1. Correlation between SCW-induced joint pain and inflammation was weak and non-significant. Specific Spearman coefficient values (r_s) and p values between pain behavior parameters - max contact max intensity, print area and single stance on the affected (right) hind limb, static weight bearing (incapacitance)– and arbitrary score of inflammation. Inflammation with proportional association to pain indicated by a positive coefficient; r_s close to zero indicating no association between parameters; and negative correlations indicated inversely proportional association between inflammation and pain behavior. N=7 males and 8 females per SCW group.

	acute (d1)			
	Male		Female	
	rs	p value	rs	p value
max contact max intensity	0.187	0.688	0.289	0.488
print area	0.281	0.542	0.192	0.648
single stance	0.056	0.905	0.192	0.648
static weight bearing	-0.374	0.408	0.577	0.134

	pre-sensitized (d8)			
	Male		Female	
	rs	p value	rs	p value
max contact max intensity	0.116	0.805	0.589	0.124
print area	0.231	0.618	0.430	0.288
single stance	0.386	0.393	0.258	0.538
static weight bearing	0.347	0.445	-0.160	0.706

Table S2. Synovial inflammatory factors abbreviations and full names

	Abbreviation	Full name
**Receptors**		
	Tlr2	Toll-like receptor 2
	Tlr4	Toll-like receptor 4
**Alarmins**		
	S100a8	S100 calcium-binding protein A8
	S100a9	S100 calcium-binding protein A9
**Chemokines**		
	Ccl2 (MCP-1)	C-C motif chemokine 2
	Cxcl1 (KC)	C-x-C motif chemokine ligand 1
**Cytokines**		
	Il1b	Interleukin-1 beta
	Tnfa	Tumor necrosis factor alfa
	Il6	Interleukin-6
	Il10	Interleukin-10
**Redox signaling**		
	Cyba	Cytochrome b-245, alpha polypeptide
	Cybb (NOX2)	Cytochrome b-245, beta polypeptide
	Ncf4	Neutrophil cytosolic factor 4
	Nos2	Nitric oxide synthase, inducible
**Neurotropic factor**		
	Ngf	Nerve growth factor
**Immune cells**		
	Emr1 (F4/80)	EGF module-containing mucin-like receptor
	Cd86	Cluster of Differentiation 86
**Pro-resolution**		
	Gas6	Growth arrest-specific protein 6

Table S3 Correlation of synovial inflammatory factors to pain behavior exhibited different profiles between sexes. Specific Spearman coefficient values (r_s) and p values between weight bearing asymmetry and synovial genes expressed during acute SCW arthritis. Genes were sorted by stronger proportional association with pain based on the male d8 group. Inflammatory genes with proportional association to pain are indicated by a positive coefficient; r_s= 0, representing no association between parameters; while negative correlations indicate inversely proportional association between synovium gene expression and pain behavior. *p<0.05 and **p<0.01. N=7 males and 8 females per SCW group.

Male d1

Gene	rs	p value
Gas6	-0.714	0.071
Cyba	0.250	0.589
Il6	0.464	0.294
Cd86	-0.143	0.760
Cxcl1	0.393	0.383
Ngf	0.429	0.337
S100a8	-0.143	0.760
S100a9	-0.250	0.250
Tlr2	-0.179	0.702
Arg1	-0.607	0.607
Cybb	-0.357	0.432
Emr1	-0.143	0.760
Il10	0.179	0.702
Ncf4	-0.607	0.607
Ccl2	-0.250	0.589
Tnfa	-0.429	0.337
Tlr4	0.071	0.879
Il1b	-0.393	0.383
Nos2	-0.714	0.071

Female d1

Gene	rs	p value
Gas6	-0.119	0.779
Cyba	0.452	0.260
Il6	-0.381	0.352
Cd86	0.144	0.734
Cxcl1	-0.120	0.778
Ngf	0.000	1.000
S100a8	0.405	0.320
S100a9	0.491	0.217
Tlr2	0.095	0.823
Arg1	0.143	0.736
Cybb*	0.762	0.028
Emr1	0.238	0.570
Il10	0.024	0.955
Ncf4	0.683	0.062
Ccl2	0.143	0.736
Tnfa	0.252	0.548
Tlr4	0.381	0.352
Il1b	0.214	0.610
Nos2	-0.643	0.086

Male d8

Female d8

Gene	rs	p value
Gas6**	0.929	0.003
Cyba*	0.857	0.014
Il6*	0.821	0.023
Cd86	0.750	0.052
Cxcl1	0.750	0.052
Ngf	0.679	0.094
S100a8	0.679	0.094
S100a9	0.679	0.094
Tlr2	0.679	0.094
Arg1	0.607	0.148
Cybb	0.607	0.148
Emr1	0.571	0.180
Il10	0.571	0.180
Ncf4	0.571	0.180
Ccl2	0.464	0.294
Tnfa	0.393	0.383
Tlr4	0.321	0.482
Il1b	0.179	0.702
Nos2	0.143	0.760

Gene	rs	p value
Gas6	0.071	0.879
Cyba	0.000	1.000
Il6	0.310	0.456
Cd86	-0.048	0.911
Cxcl1	0.333	0.420
Ngf	-0.024	0.955
S100a8	0.310	0.456
S100a9	0.286	0.493
Tlr2	0.429	0.289
Arg1	0.381	0.352
Cybb	0.310	0.456
Emr1	0.476	0.233
Il10	0.143	0.760
Ncf4	0.357	0.385
Ccl2	0.262	0.531
Tnfa	0.575	0.136
Tlr4	0.536	0.215
Il1b	0.429	0.289
Nos2*	0.738	0.037
